# Pancreaticoduodenal and choledochal hemangiomatosis with vascular variation in a child: a rare disease with challenge starts from diagnosis—a case report

**DOI:** 10.1186/s12957-022-02737-5

**Published:** 2022-09-07

**Authors:** Daguang Tian, Hong Zhu, Xiaoping Wei

**Affiliations:** Department of HPB Surgery, The Second Affiliated Hospital of Kunming Medical University, 374 Burma Road, Kunming, 650101 Yunnan China

**Keywords:** Hemangiomatosis, Pancreas, Duodenum, Choledoch, Vascular variation, Misdiagnosis, Case report

## Abstract

**Background:**

Visceral hemangiomatosis is a benign tumor (rarer than hemangioma) that has not been reported to occur in the pancreas, duodenum, or choledoch. It can be easily confused with other pancreatic tumors or choledocholithiasis. Herein, we describe a case of a child with pancreaticoduodenal and choledochal hemangiomatosis and the key characteristics for the accurate diagnosis of pancreatic tumors based on previous reports and our findings.

**Case presentation:**

We report a case of a 2-year and 9-month-old child who presented with repeated and fluctuating jaundice for 3 months with a history of endoscopic stone removal in a local hospital, following the diagnosis of choledocholithiasis. An abdominal computed tomography revealed a previously undiagnosed pancreatic head tumor and celio-mesenteric trunk (a rare vascular variation). This was misdiagnosed as a pancreatic neuroendocrine tumor. Since the patient’s parents refused FNA biopsy and insisted on surgery, pancreaticoduodenectomy was performed; however, postoperatively, the child was correctly diagnosed with pancreaticoduodenal and choledochal hemangiomatosis. Although the patient was in good condition and had gained 4 kg in weight 3 months postoperatively, pancreaticoduodenectomy could have been avoided if an accurate diagnosis had been established before or during the operation.

**Conclusion:**

Our report highlights the difficulty in diagnosing visceral hemangiomatosis. Radiologists, endoscopists, and surgeons should consider this possibility in cases of repeated and fluctuating jaundice that cannot be explained by choledocholithiasis alone.

## Background

Tumors of the pancreatic head, duodenum, and choledoch cause obstructive jaundice, and surgical treatment is usually warranted. Some of these rare tumors remain undiagnosed or are easily misdiagnosed. Hemangioma is a common benign tumor that occurs mostly in the liver and spleen; however, pancreatic hemangioma only accounts for 0.1% of pancreatic tumors [1]. Hemangiomatosis, which is different from hemangioma, shows signs of diffuse and persistent vascular proliferation mostly on the skin and in the muscles. Pancreatic hemangiomatosis has an extremely low incidence (only one case has been reported till date) [2]. Furthermore, two studies on duodenal hemangiomatosis and no study on choledoch have been reported since 1980 [3, 4]. Herein, we report a case and provide a literature review of hemangiomatosis occurring at three sites simultaneously, which has not been previously reported.

## Case presentation

**Fig. 1:**
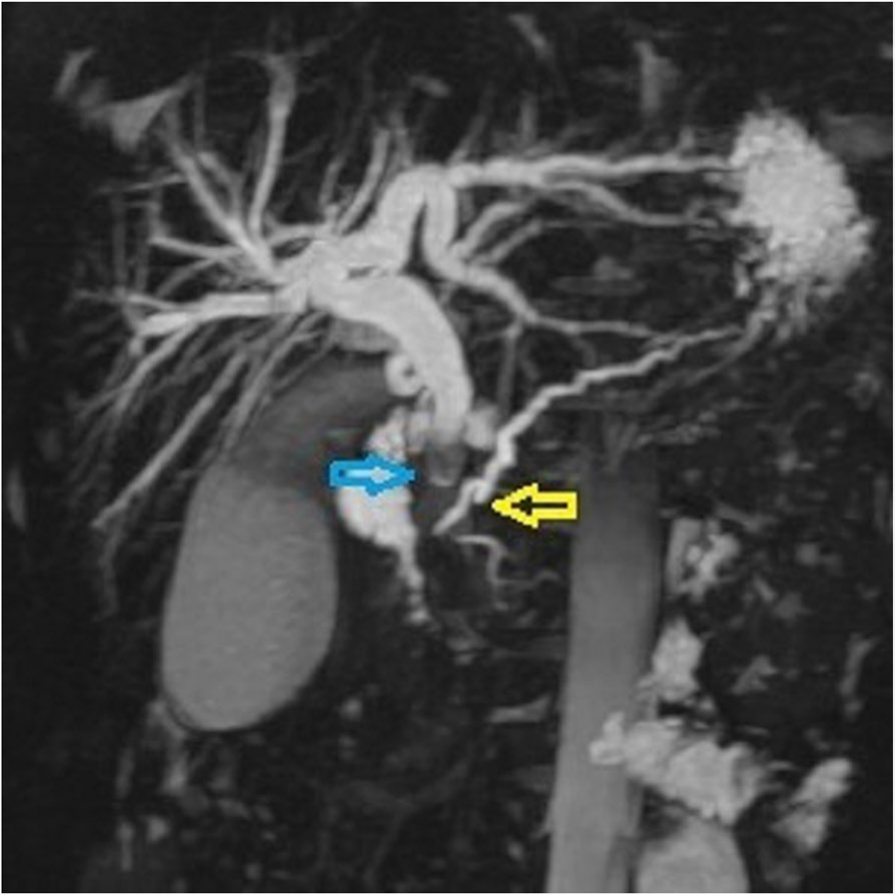
Magnetic resonance cholangiopancreatography (MRCP) shows hepatobiliary duct dilation and stones in the common bile duct (blue arrow). The main pancreatic duct dilated irregularly (yellow arrow)

**Fig. 2:**
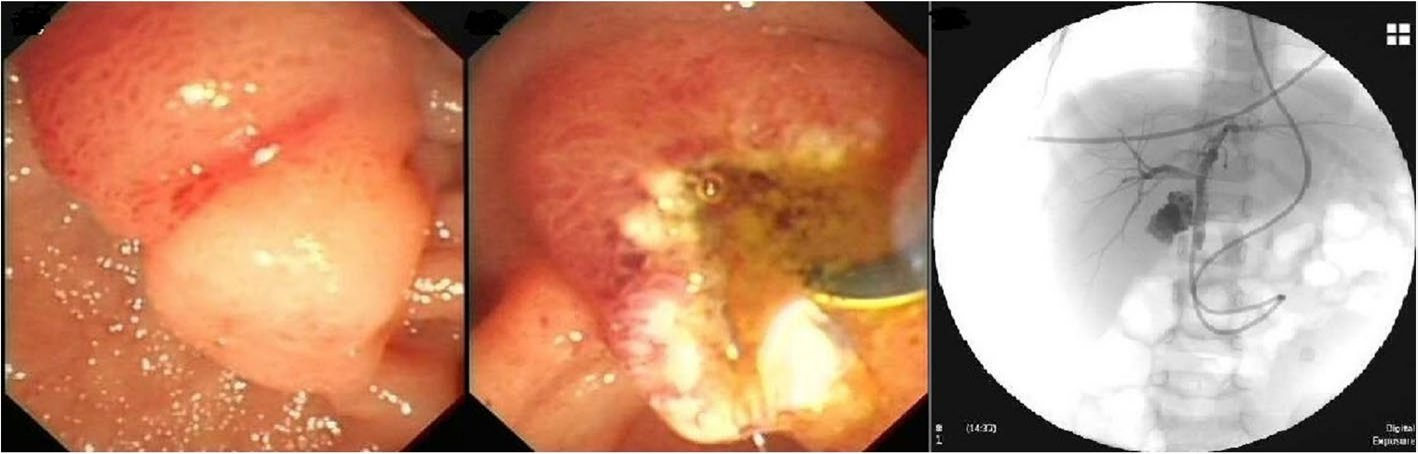
Endoscopic retrograde cholangiopancreatography (ERCP) shows enlarged duodenal papilla (**a**) and silt-like stones were removed after sphincterotomy (**b**). Endoscopic nose biliary drainage tube cholangiography shows stenosis at the lower end of the common bile duct

**Fig. 3:**
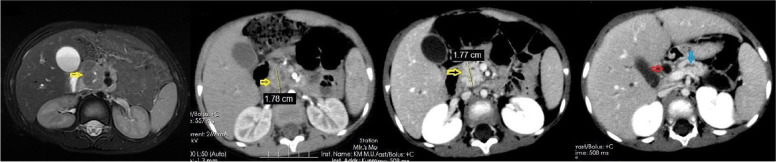
Magnetic resonance imaging (**a**) (in local children’s hospital, but not reported at that time) and computed tomography scan (**b**) (in our hospital) show a tumor with an inhomogeneous density at the pancreatic head (yellow arrow). In the delayed phase, the mass shows obvious enhancement (**c**) with the dilated common bile duct (red arrow) and pancreatic duct (blue arrow, **d**)

**Fig. 4:**
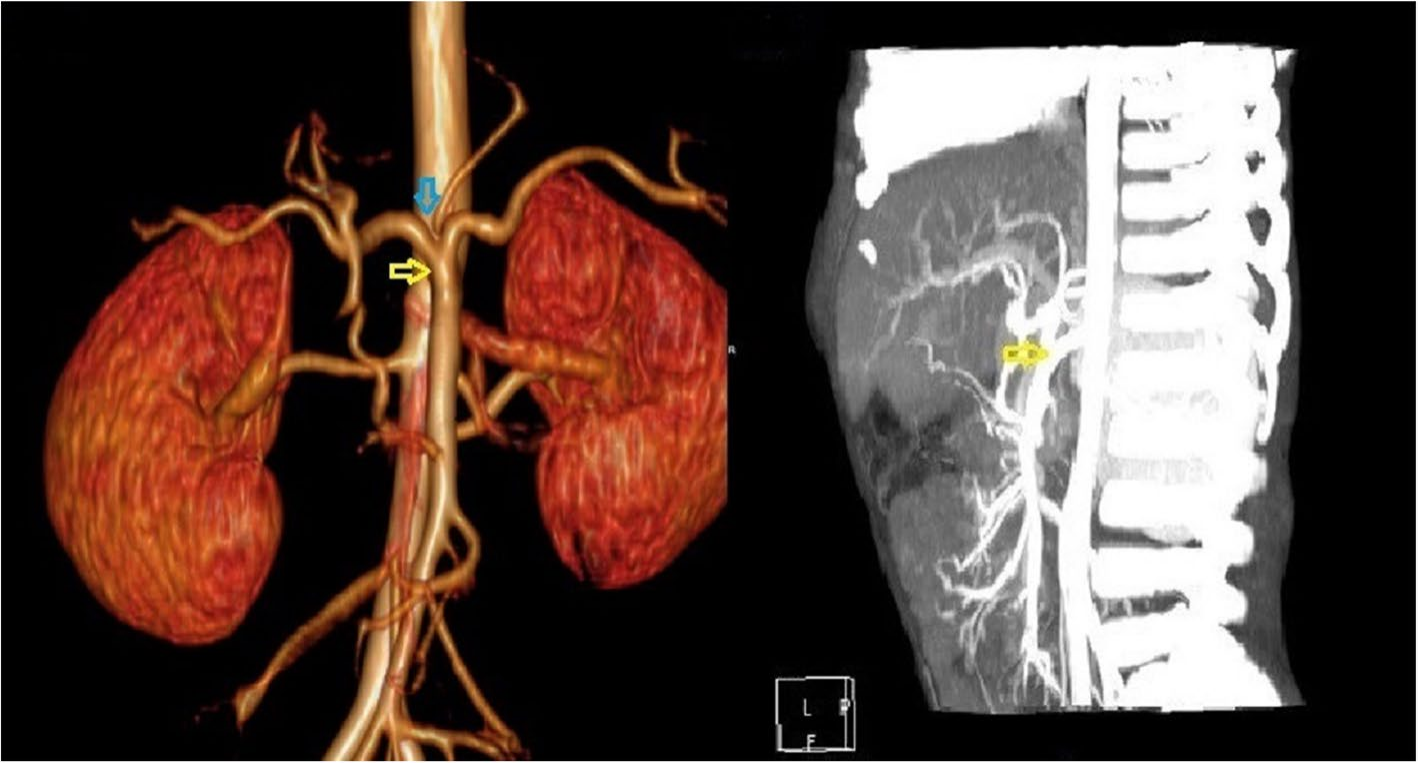
Abdominal computed tomography angiography shows that the celiac trunk and proximal superior mesenteric artery had a common trunk (CMT) (yellow arrow). The left gastric artery originated from the abdominal aorta (blue arrow)

**Fig. 5:**
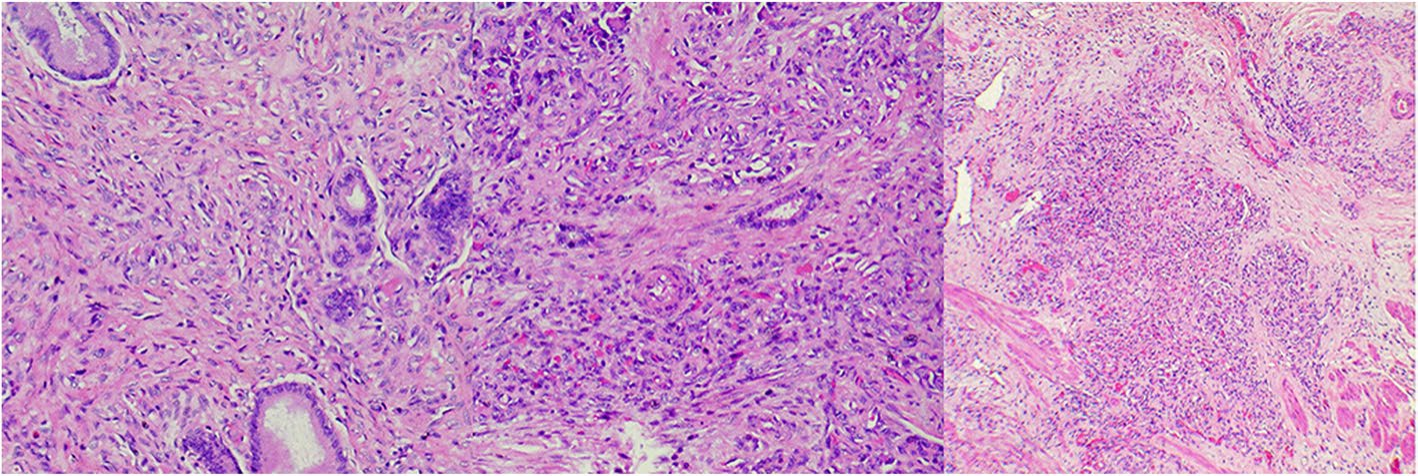
Histology shows diffuse, nodular proliferation of the capillaries in the pancreas (**a**), common bile duct (**b**), and duodenal muscle layer (**c**) with lymphocyte infiltration

**Fig. 6:**
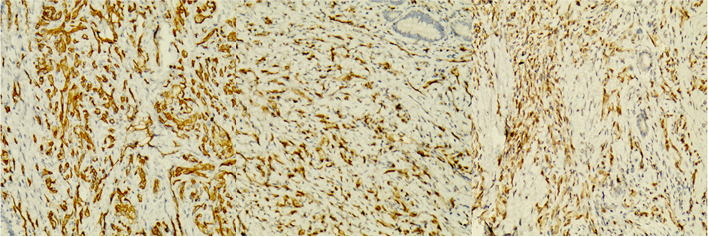
CD31(+) (**a**), ERG(+) (**b**), and FLI-1(+) (**c**) in immunohistochemistry

**Fig. 7:**
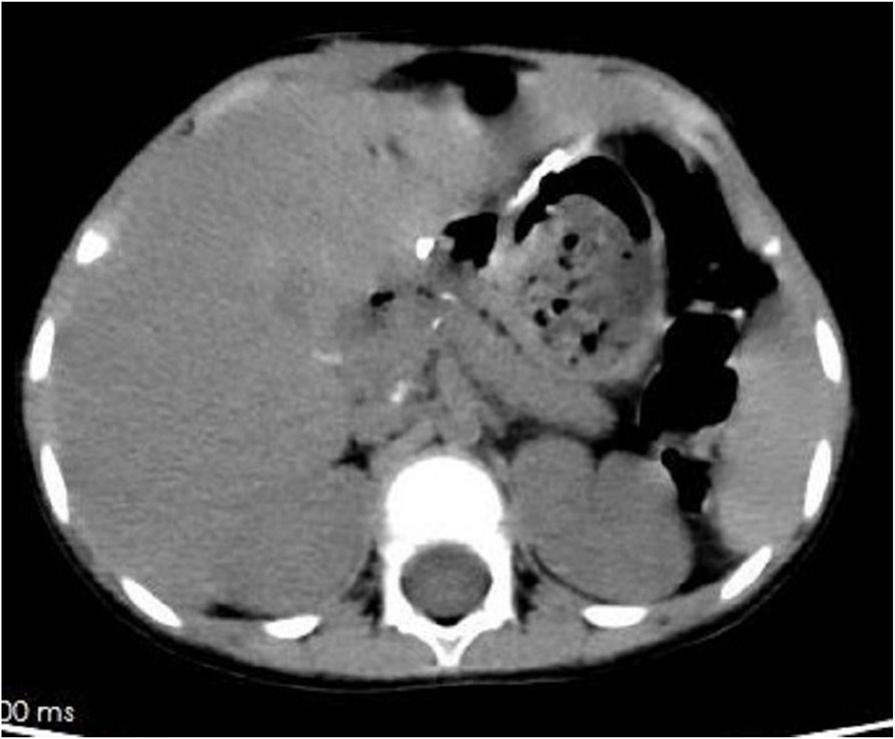
Abdominal CT showed no recurrence 3 months after the operation

A male patient aged 2 years and 9 months with abdominal pain and sclera jaundice was referred to a municipal children’s hospital from a county hospital. He was diagnosed with choledocholithiasis by magnetic resonance imaging (MRI) with liver dysfunction (Fig. 1, Table 1) on December 4, 2020. Because endoscopic retrograde cholangiopancreatography (ERCP) could not be performed in the municipal hospital, he was referred to our hospital and underwent endoscopic stone removal on December 17, 2020 (Fig. 2). Thereafter, jaundice resolved rapidly (Table 1). However, the patient reported back to our institute with recurrent jaundice on February 19, 2021. Physical examination revealed severe jaundice, evidenced by the color of his skin and sclera, and his liver was palpable 4 cm under the right rib. Liver function tests showed significantly elevated levels of alkaline phosphatase, gamma-glutamyl transpeptidase, total bilirubin, and direct bilirubin (Table 1). Pancreatic neuroendocrine tumor (panNET) was diagnosed based on abdominal computed tomography (CT) scan (Fig. 3). Also, the CT scan showed that the left gastric artery (LGA) originated from the aorta with a celio-mesenteric trunk (CMT) (Fig. 4), and the parents refused FNA biopsy, so pancreaticoduodenectomy (PD) was performed on March 1, 2021. Unfortunately, the postoperative diagnosis was pancreaticoduodenal and choledochal hemangiomatosis based on pathology findings (Fig. 5). Furthermore, immunohistochemistry showed CD34(+), ERG(+), and FLI-1(+) markers (Fig. 6). Liver function improved dramatically on postoperative day 2 and was almost normal by day 10 (Table 1). Three months later, the patient had no recurrent jaundice and had gained 4 kg in weight. Additionally, abdominal CT showed no recurrence (Fig. 7).

**Table 1:** Fluctuation of liver function

	December 4, 2020	December 18, 2020	February 20, 2021	March 3, 2021	March 11, 2021
ALT (U/L)	131	34	162	49	34
ALP (U/L)	639	255	1341	328	275
GGT (U/L)	827	77	1204	233	123
TBIL (μmol/L)	164	31	148	83	26
DBIL (μmol/L)	134	27	113	69	21

*ALT* alanine transferase, *ALP* alanine phosphatase, *GGT* gamma glutamyl transferase, *TBIL* total bilirubin, *DBIL* direct bilirubin

## Discussion and conclusions

Visceral hemangioma is a common benign tumor, which occurs mostly in the liver. Hemangioma is a vascular malformation, which can be divided into sclerosing hemangioma, hemangioendothelioma, capillary hemangioma, and cavernous hemangioma according to the amount of fibrous tissue it contains. Hemangiomatosis is an uncommon benign vascular malformation representing errors in normal vascular morphogenesis. Different from hemangioma, it is characterized by a diffuse excess of dilated, capillary-sized flat endothelial-lined blood vessels in normally vascular tissue. The cause of the disease is unknown; however, it may be formed by local proliferation and differentiation of some hemangioblasts that detach from the vascular network in the embryonic developmental period. Recent studies have shown that its production may be related to vascular production stimulating factors, such as basic fibroblast growth factor and vascular endothelial growth factor (VEGF) [5, 6]. Hemangiomatosis is commonly observed on the skin, subcutaneous tissue, muscle, and bone, and visceral hemangiomatosis is a comparatively rare disease. One study about its occurrence in the pancreas [2] and two in the duodenum [3, 4] have been reported (Table 2); however, no study regarding its occurrence in the bile duct has been reported. To our knowledge, this is the first report of hemangiomatosis occurring simultaneously in three sites.

**Table 2:** Cases of hemangiomatosis on the pancreas and duodenum

Author	Presentation	Image	Clinical diagnosis	Location	Treatment	Histology
Tetsuya et al. [2]	Abdominal pain	US, CT	Angiomatosis	Pancreas	PD	Angiomatosis
Ikeda et al. [3]	Fainting Diaphoresis Melena	Endoscopy	Hemangiomatosis	Duodenum	Duodenal resection	Hemangiomatosis
Lakhkar et al. [4]	Bilious vomiting	Barium meal CT	Hemangiomatosis	Duodenum jejunum	Gastro-jejunostomy Oral corticosteroid	-

*US* ultrasound, *CT* computed tomography, *PD* pancreaticoduodenectomy

Visceral hemangiomatosis may be asymptomatic or subside spontaneously. The symptoms are mostly related to the location, such as abdominal pain, melena, and anemia. It can be accompanied by some rare clinical syndromes such as Klippel–Trenaunay syndrome, Kasabach–Merritt syndrome, and Von Hippel–Lindau syndrome [7–9]. The present case was not related to these syndromes, and obstructive jaundice was the initial sign. Endoscopic stone removal was performed after the diagnosis of choledocholithiasis based on MRI, and the radiologists in the local hospital did not observe a tumor in the pancreas.

In retrospect, choledocholithiasis was likely formed by bile duct obstruction, caused by a combination of pancreatic head compression and proliferation of diffuse hemangioma in the duodenum and the lower end of the bile duct. In our hospital, an unclear boundary lesion was observed on CT, and the case was misdiagnosed as panNET. The misdiagnosis may be attributed to the inexperience of the radiologists or to the poor accuracy of CT or MRI for diagnosis. Hence, more attention was needed to differentiate hemangiomatosis from more common pancreatic tumors, such as panNET, cavernous hemangioma, and Kaposi’s hemangioendothelioma, which can be identified by key characteristics.

panNET accounts for 1.3% of all pancreatic tumors [10]. On MRI, it shows a high signal with cystic degeneration and areas of necrosis of varying scope. On enhanced scanning, the solid components generally exhibit uneven enhancement, whereas the necrotic areas exhibit no enhancement.

Cavernous hemangioma is mainly composed of enlarged vascular cavities with a complete capsule. CT scans show an analogous round, solid-cystic mass with mixed density. After enhancement, it exhibits obvious marginal nodular enhancement in the arterial phase and filling progressively from margins to the center in the venous phase.

Histologically, Kaposi’s hemangioendothelioma has the characteristics of both hemangioma and Kaposi sarcoma and is accompanied by Kasabach–Merritt syndrome [8]. MRI T1 image shows low or iso-signal lump-like soft tissue with unclear boundaries. T2 image shows a diffuse high signal after enhancement, and the boundary is not clear in the surrounding tissues. In this case, a CT scan revealed an enlarged pancreatic head with high density and an unclear boundary shadow. In the arterial phase, the mass was unevenly enhanced, indicating that it is difficult to distinguish hemangiomatosis from other common tumors based on imaging examination alone.

Endoscopy can help find duodenal hemangiomatosis by locating dilated blood vessels in the mucosal layer and blue hemangioma under the mucosa. Two previously reported cases [3, 4] showed blue hemangioma on gastroscopy. In our case, no abnormality was observed, except for an enlarged papilla, indicating that endoscopy is less helpful for cases located in deeper layers (such as the muscle).

For pancreatic tumors, fine-needle aspiration (FNA) biopsy was suggested by some scholars [11]. However, if the lesion is a hemangioma, the risk of bleeding is high. If the tumor is malignant, there is a possibility of needle tract metastasis. In some cases, accurate diagnosis cannot be established due to scarce specimens or inexperienced pathologists. Thus, diagnosing pancreatic tumors requires joint input from radiologists, endoscopists, and surgeons in a case of repeated and fluctuating jaundice that is not caused by choledocholithiasis alone.

We believe that histology is the most accurate method of diagnosing hemangiomatosis. Histologically, there are two types of hemangiomatosis. The first is a mix of veins, cavernous blood vessels, and capillaries, with thick vascular walls and small vessels forming clusters at the bulge. The other type is diffuse hyperplasia of capillaries, which appears as hyperplastic small to medium-size vessels with irregular shapes under a light microscope: compared with the first type, vascular walls are thin and may show lymphocyte infiltration. Usually, vascular markers, CD31F and CD34, are positive.

A notable feature of the present case is fluctuating jaundice. Jaundice was the first symptom exhibited by the patient, with the total bilirubin level reaching approximately 164 μmol/L. Although it decreased dramatically (31 μmol/L) after endoscopic stone removal, jaundice reappeared quickly (148 μmol/L). Therefore, fluctuating jaundice can be considered the main factor that necessitated surgery. Obstructive jaundice is often characterized by stones in the bile duct or tumors in the pancreatic head, and fluctuating jaundice indicates the presence of lesions in the ampulla of Vater [12, 13].

Surgical intervention may be needed once acute gastrointestinal hemorrhage or obstructive jaundice appears [3, 4]. However, surgery sometimes fails to remove lesions completely due to their multicentric character, and internal medicine may be needed. Studies have shown that among children with hemangiomatosis treated with corticosteroids, about one-third responded well, whereas one-third gained no benefit [5]. Although interferon-alpha was administered to severely ill patients, it caused severe side effects [14]. In recent years, anti-VEGF drugs [15], such as ranibizumab and bevacizumab, have been used clinically.

In our case, PD was performed after the diagnosis of panNET because the patient’s parents refused FNA biopsy and insisted on surgery. So far, only two cases of obstructive jaundice caused by pancreatic head hemangioma have been reported [16, 17] (aged 5 months and 2 years), and neither of the tumors was removed. Based on the limited literature available, PD or any operation may not have been the best choice for our patient, although his parents were satisfied with his rapid recovery. Better communication with patients’ parents and insistence on intraoperative biopsy could have provided other treatment options such as drug therapy, simple T-tube drainage, or cholangiojejunostomy. Although the age of the youngest patient reported to have undergone PD, with satisfactory follow-up outcomes, was 11 months [18], we believe that an accurate preoperative diagnosis could have provided several other treatment options.

Furthermore, we observed rare vascular variations, as the CMT and LGA originated from the abdominal aorta. There are four trunks on the dorsal side of the original abdominal aorta during the embryonic period; they include LGA, hepatic artery, splenic artery, and superior mesenteric artery from top to bottom, and there are anastomotic arteries between them longitudinally. In most cases, these anastomotic arteries between the third and fourth trunks are interrupted during development, the celiac trunk and superior mesenteric artery (SMA) are separate, and different openings are formed. However, an anastomotic artery can persist and may be the basis of CMT development with an incidence of less than 1% [19].

Changes in the origin of the celiac trunk can lead to variations in the LGA. Overall, the variation rate of LGA is approximately 2.8% [18], and LGA originating from the abdominal aorta with CMT has an incidence of 1.6% [20]. Vascular variation is mostly caused by abnormal embryonic development, and the cause of hemangiomatosis may also be related to the abnormal development of the blood vessels during the embryonic period. The relationship between them and the involvement of genetic mutation is unknown. Additionally, there are no reports about hemangiomatosis complicated with vascular variation, and the patient’s parents refused genetic screening. Hence, future studies elucidating these relationships are warranted.

Although hemangiomatosis is a rare disease, if we can improve the level of reading imaging data before surgery or strengthen communication with the patient’s parents to perform FNA, it may be possible to establish an accurate diagnosis before surgery. Therefore, the child may avoid surgical trauma and receive better treatment. Furthermore, we consulted with an outside pathologist who recommended that this lesion should be classified as a pediatric pancreatic capillary hemangioma rather than hemangiomatosis, which is a lesion described by the WHO as primarily located in the lungs. Hence, further consideration of differential diagnosis is warranted to improve our understanding of this disease.

## Data Availability

The datasets used and/or analyzed during the current study are available from the corresponding author on reasonable request.

## Abbreviations

CMT: Celio-mesenteric trunk
CT: Computed tomography
ERCP: Endoscopic retrograde cholangiopancreatography
FNA: Fine-needle aspiration
LGA: Left gastric artery
MRI: Magnetic resonance imaging
panNET: Pancreatic neuroendocrine tumor
PD: Pancreaticoduodenectomy
VEGF: Vascular endothelial growth factor
